# Phenolic Composition, Wound Healing, Antinociceptive, and Anticancer Effects of *Caralluma europaea* Extracts

**DOI:** 10.3390/molecules28041780

**Published:** 2023-02-13

**Authors:** Fatima Ez-Zahra Amrati, Mohamed Chebaibi, Renata Galvão de Azevedo, Raffaele Conte, Meryem Slighoua, Ibrahim Mssillou, Sotirios Kiokias, Alice de Freitas Gomes, Gemilson Soares Pontes, Dalila Bousta

**Affiliations:** 1 Laboratory of Biotechnology, Health, Agrofood and Environment (LBEAS), Faculty of Sciences Dhar El Mehraz, Sidi Mohamed Ben Abdellah University, Fez 30000, Morocco; 2 Biomedical and Translational Research Laboratory, Faculty of Medicine and Pharmacy of the Fez, University of Sidi Mohamed Ben Abdellah, Fez 30000, Morocco; 3 Laboratory of Virology, National Institute of Amazonian Research (INPA), Manaus 69067-375, Brazil; 4 Research Institute on Terrestrial Ecosystems (IRET)-CNR, 80131 Naples, Italy; 5 Laboratory of Natural Substances, Pharmacology, Environment, Modeling, Health and Quality of Life (SNAMOPEQ), Faculty of Sciences Dhar El Mahraz, Sidi Mohamed Ben Abdellah University, Fez 30000, Morocco; 6 European Research Executive Agency (REA), 1210 Bruxelles, Belgium; 7 Post-Graduate Program in Hematology, School of Health Sciences, University of the State of Amazonas, Manaus 69050-010, Brazil

**Keywords:** *Caralluma europaea*, phenolic compounds, UHPLC, wound healing, antinociceptive activity, leukemia, hepatocellular carcinoma, molecular docking

## Abstract

*Caralluma europaea* (Guss.) is an important medicinal plant widely used in Morocco for various traditional purposes. Our work aimed to evaluate the phenolic composition, wound healing, antinociceptive, and anticancer activities of *C. europaea* extracts. Moreover, this study assessed the beneficial effect of *C. europaea* phytocompounds on the TRADD, cyclooxegenase-2, Wnt/β-catenin, and tyrosine kinase signaling pathways. The wound healing effect of *C. europaea* formulations against skin burn was evaluated for 21 days. The cytotoxic effect of the *C. europaea* extracts was evaluated against human leukemic (K562 and HL60) and liver cancer cell lines (Huh-7) using the MTT test. All the phytoconstituents identified by UHPLC in the polyphenols were docked for their inhibitory power on protein casein kinase-1, glycogen synthase kinase-3-β, cyclooxegenase-2, tyrosine kinase, and TRADD. Luteolin and kaempferol are the main compounds identified in *C. europaea* polyphenols. The group treated with polyphenols showed the greatest wound contractions and all tested extracts presented a significant antinociceptive effect. Polyphenols showed a remarkable antitumoral activity against the K562, HL60 and Huh-7 cell lines. Saponins exerted an important cytotoxic effect against the Huh-7 cell line, whereas no cytotoxicity was observed for the hydroethanolic and flavonoids extracts. Hesperetin and trimethoxyflavone presented the highest docking G-score on tyrosine kinase and cyclooxygenase, respectively.

## 1. Introduction

The majority of the world’s population have become more dependent on herbal medicine for their first-line of treatment due to the negative effects of conventional modern medicine. Skin burns and cancerous diseases cause intense pain, affecting the well being of patients more than the diseases with which they are diagnosed. Plant’s ability to cure these diseases motivated scientists to evaluate their biological activities [1].

Hepatocellular carcinoma is one of the common prevalent human cancers worldwide with an extremely bad prognosis. Various scientific studies reported that the development of hepatocellular carcinoma is a slow process, characterized by several genetic and epigenetic abnormalities that result in the activation of oncogenes or inactivation of tumor suppressor genes [2]. Cancer blood or leukemia is a varied group of hematologic malignancies characterized by unusual growth of immature lymphoid cells [3]. Approximately 60,530 new leukemia cases were detected in 2020, among which 19,940 new cases were diagnosed with acute myeloid leukemia and 8450 with chronic myeloid leukemia. A total of 23,100 people are predicted to die from leukemia in 2020 [4]. Studies have shown that leukemia and its treatment are often associated with fatigue, pain and dermatological disorders. Patients with chronic leukemia commonly have high incidence of dermatological problems such as dermatitis, drug exanthems, exaggerated insect bite reactions, bacterial, viral, and fungal infections [5]. Pain is also a frequently reported sign among patients with leukemia and liver cancer, which can affects the quality of life of patients [6].

*C. europaea* is one of plants used in Moroccan traditional medicine to treat various diseases including inflammatory diseases, pain, and cancer. Previous research works have revealed the antidiabetic, antimicrobial, antioxidant, anti-inflammatory, and hepatoprotective effects of *C. europaea* [7,8,9]. These pharmacological activities have been explained by the phytochemicals already identified in the plant such as luteolin, gallic acid, hesperetin, quercetin, myricetin, ferulic acid, salicylic acid, naringenin-7-glucoside [10].

To the best of our knowledge, there have been no scientific studies on the burn-healing or cytotoxic activities on leukemia and hepatocarcinoma of *C. europaea* extracts. The antioxidant, antibacterial, anti-inflammatory, and anticancer effects on pancreatic cancer of the *C. europaea* extracts already demonstrated in our previous studies inspired us to test the other pharmacological effects of this plant on the treatment of burns, leukemia, hepatic cancer, and pain.

Our study aimed (i) to perform the chemical characterization of *C. europaea* polyphenolic extract, (ii) to evaluate the wound healing on skin burn, the antinociceptive on contortions and writhes induced by acetic acid and formalin injections, respectively, and the anticancer effects of *C. europaea* fractions against leukemia (K562 and HL60) and human liver hepatocellular carcinoma (Huh-7) cell lines using the MTT test, and (iii) to determine the molecules that have been identified in our plant, and that can have a major effect on casein kinase-1, glycogen synthase kinase-3β, cyclooxygenase-2, tyrosine kinase, and TRADD by using molecular docking.

## 2. Results

### 2.1. Chemical Analysis of C. europaea Polyphenolic Extract

The LC-MS/MS analysis of *C. europaea* polyphenolic extract revealed the presence of several phenolic compounds. The main identified flavonoids are kaempferol, luteolin, and kaempferol-3-*O*-hexose deoxyhexose, and the main identified phenolic acids are trans-ferulic acid and syringic acid. The AUC of the identified phytocompounds is presented in Table 1.

Identification of isomers with the same molecular weight was confirmed by analyzing their characteristic fragmentation patterns and the main product ions observed in the MS–MS spectrum as reported by Li Z.H. et al. [11]. In particular, 609.1 > 284 and 609.1 > 255 were used for kaempferol-3-*O*-glucose, 609.1 > 300 was adopted for quercetin-3-*O*-hexose deoxyhexose, 447.1 > 315 and 447.1 > 300 were utilized for isorhamnetin-7-*O*-pentose, and 447.1 > 285 and 447.1 > 151 were used for luteolin 7-*O*-glucoside. Moreover, the differentiation of the ferulic acid from the trans-ferulic acid was obtained according to their selective sensibility at the ionization’s polarity. In fact, the presence of the ferulic acid was confirmed according to the method of Seraglio et al. [12], which analyzed the [M + H]^+^ adduct of this compound (195 > 176.9; 195 > 89). For the trans-ferulic acid, instead, the negative adduct 193 > 133.8 was selected for confirmation [13].

### 2.2. Cytotoxicity of C. europaea on Human Leukemia and Hepatocellular Carcinoma

The *C. europaea* extracts were tested for their anticancer effect against three cancer cell lines: K562, HL60, and Huh-7. As shown in Table 2, polyphenols and saponins were the most potent extracts in inhibiting cancer cells. The polyphenolic extract was able to inhibit the proliferation of the K562 (IC_50_ = 36.47µM), HL60 (IC_50_ = 24.07 µM) and Huh-7 cancerous cell lines (IC_50_ = 53.77 µM). Saponins were able to inhibit the proliferation of the Huh-7 cancerous cell lines (IC_50_ = 50.14 µM).

**Figure 1:**
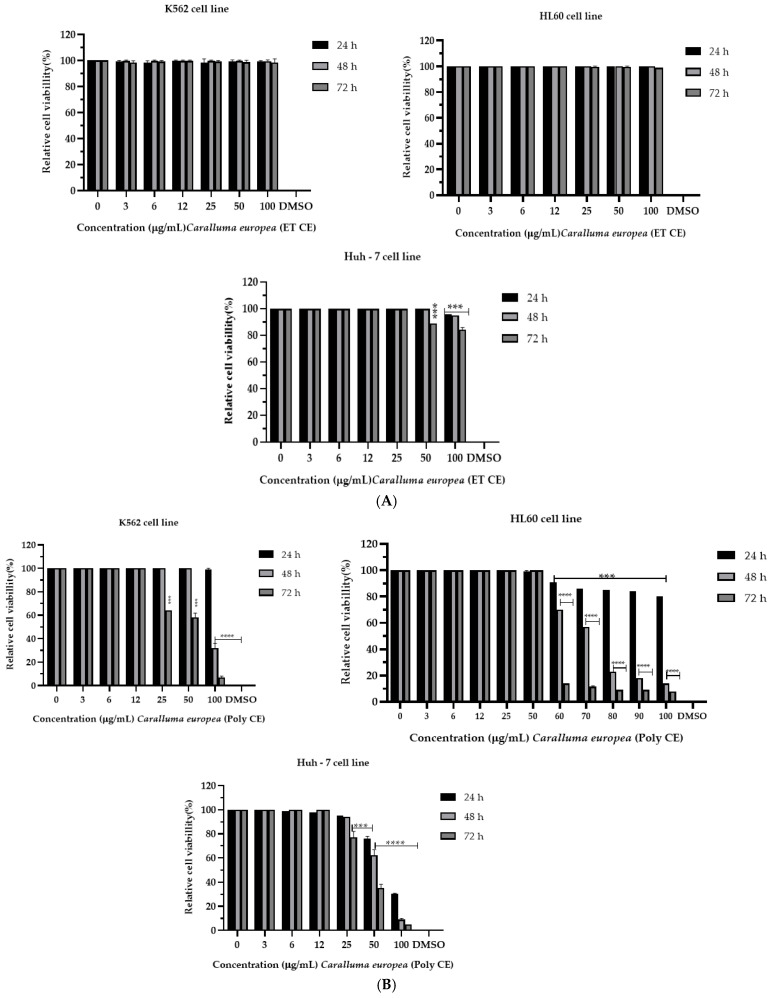

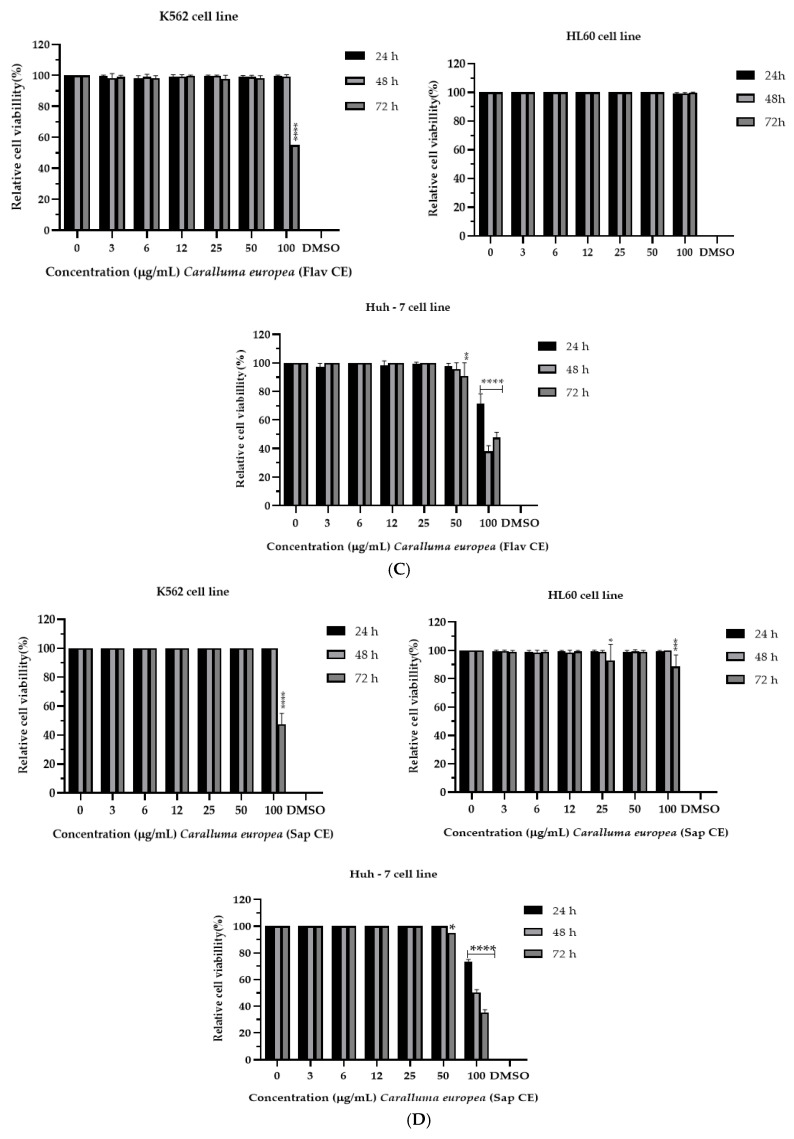
Cytotoxicity of the *C. europaea* extracts for K562, HL60, and Huh-7 cell lines. (**A**): Hydroethanolic extract; (**B**): Polyphenolic extract; (**C**): Flavonoids extract; (**D**): Saponins extract, after 24, 48, and 72 h of treatment with different concentrations of the *C. europaea* extracts. The wavelength of 570 nm was used to measure the absorbance. The results are expressed as mean ± SD of three experiments. The error bars represent the standard deviation of cell viability at each concentration tested. Cell viability was assessed through MTT assay. Values significantly different compared to negative control * *p* < 0.05; **: *p* < 0.01; *** *p* < 0.001; **** *p* < 0.0001.

The hydroethanolic, flavonoids, and saponins extracts of *C. europaea* showed no effect on the K562 and HL60 cell lines, at all concentrations tested, while a significant effect was observed on the Huh-7 cell line at the concentration of 100 µg/mL (Figure 1A–C).

Regarding the K562 cell line, the *C. europaea* polyphenolic extract reduced cell viability to 40% at doses of 25 and 50 µg/mL, and to more than 90% at a dose of 100 µg/mL after 72 h of treatment (Figure 1D).

For the HL60 cell lines, the polyphenolic extract reduced cell viability to more than 80% at doses of 80, 90, and 100 µg/mL after 48 h of treatment. For the Huh-7 cell line, the CE polyphenols reduced the cell viability to 70% after 24 h of treatment, and to more than 90% after 48 h and 72 h of treatment, at the dose of 100 μg/mL (Figure 1B).

Polyphenolic extract of *C. europaea* had an important cytotoxic effect against the three tested cell lines. It reduced cell viability of K562 to 40% at doses of 25 and 50 µg/mL, and to more than 90% at a dose of 100 µg/mL after 72 h of treatment. In addition, it reduced the cell viability of HL60 cell lines to more than 80% at doses of 80, 90, and 100 µg/mL after 48 h of treatment. For the Huh-7 cell line, the polyphenols reduced the cell viability to 70% after 24 h of treatment, and to more than 90% after 48 h and 72 h of treatment, at the dose of 100 μg/mL (Figure 1B).

### 2.3. Wound Healing Activity of C. europaea Extracts

**Figure 2:**
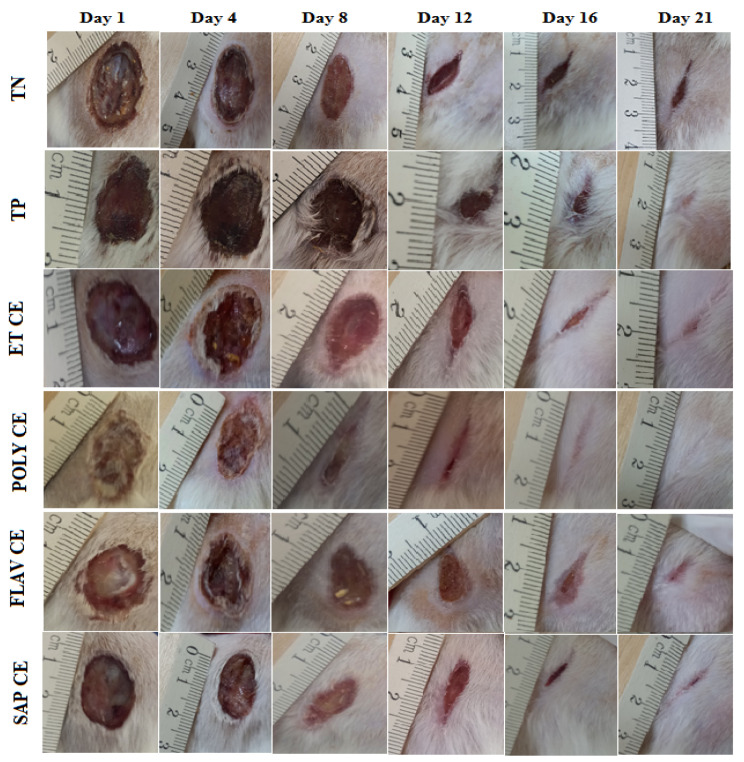
Macro-area of wounds in 1st, 4th, 8th, 12th, 16th and 21st day of experiment. CE, *C. europaea*, ET, Total extract; POLY, Polyphenols; FLAV, Flavonoids; SAP, Saponins; TP, Positive control; TN, Negative control.

Compared to the control groups, the topical application of ointments prepared from the *C. europaea* extracts significantly accelerated wound healing. Figure 2 shows the photographic development of wound healing of the *C. europaea* ointments and the vehicle groups. All of the ointments showed considerable wound closure from the first day to the 21st day. Topical use of the *C. europaea* ointments led to wound closure after 21 days. However, the wounds of the negative control (Vaseline^®^) and the positive control (Madecassol^®^) did not completely close on the 21st day of the test.

**Figure 3:**
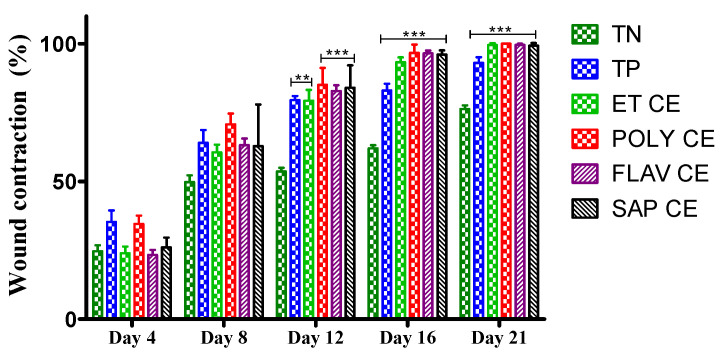
Wound contraction rate (%) on the 21 days of animal experiment. TP, Positive control; TN, Negative control ET, Total extract; POLY, Polyphenols; FLAV, Flavonoids; SAP, Saponins. Value significantly different compared to negative control: **: *p* < 0.01, ***: *p* < 0.001.

Figure 3 shows the results of wound contractions on the 4th, 8th, 12th, 16th and 21st days. The group treated with the polyphenols extract showed the best wound contractions on the 4th (34.539 ± 3.054%), 8th (70.744 ± 3.912%), 12th (85.106 ± 6.111%), 16th (96.687 ± 3.018%) and 21st day (100 ± 0.00%). On day 21, the polyphenols extract showed the highest wound contractions (100 ± 0.00%), followed by flavonoids (99.537 ± 0.463%), hydroethanolic extract (99.456 ± 0.724%), and the saponins (99.267 ± 0.976%). A wound contraction of 93 ± 2.082% was noted in the group that received Madecassol^®^ on the 21st day. The vehicle group had the lowest wound contractions (76.25 ± 1.397%), where the wounds were not fully closed.

### 2.4. C. europaea Extracts May Have a Significant Antinociceptive Effect

The antinociceptive effect of the hydroethanolic extract, polyphenols, flavonoids, and saponins of *C. europaea* was assessed using two assays in mice: acetic acid and formalin tests.

#### 2.4.1. Acetic Acid-Induced Writhing

**Figure 4:**
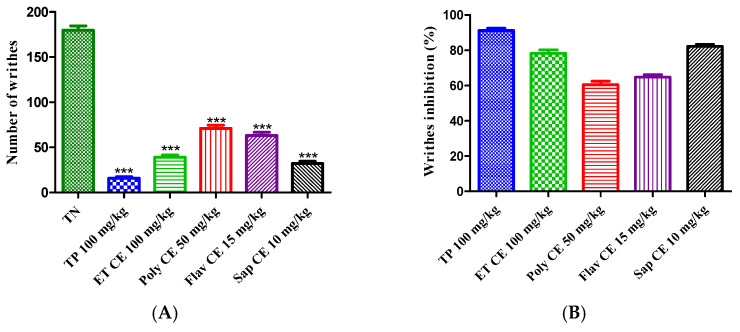
Antinociceptive activity of the *C. europaea* extracts on mice’s writhing behavior induced by acetic acid. (**A**) Number of abdominal contractions. (**B**) Inhibition of abdominal contractions in the treated groups. Values are expressed as mean ± SEM. Results are statistically different from the negative control ***: *p* < 0.001.

The acetic acid injection caused 179.700 ± 4.842 writhes in the control mice. Pretreatment with hydroethanolic extract (100 mg/kg), polyphenols (50 mg/kg), flavonoids (15 mg/kg), and saponins (10 mg/kg) of *C. europaea* decreased the number of writhes to 78.29%, 60.76%, 64.27%, and 82.19%, respectively. Interestingly, the antinociceptive effect of the saponins extract was near paracetamol at the dose of 100 mg/kg (91.28% inhibition) (Figure 4).

#### 2.4.2. Formalin-Induced Paw Licking Test

**Figure 5:**
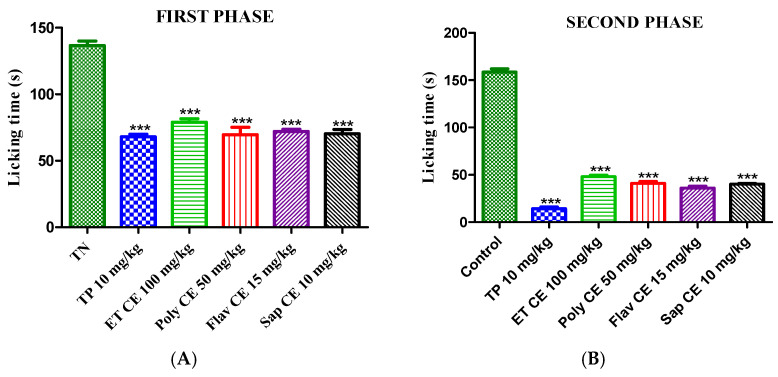
The antinociceptive activity of the *C. europaea* extracts on formalin-induced pain in mice during the first (**A**) and second (**B**) phases. Values are expressed as mean ± SEM. Results are statistically different from the negative control ***: *p* < 0.001.

Pretreatment with the *C. europaea* hydroethanolic extract (100 mg/kg), polyphenols (50 mg/kg), flavonoids (15 mg/kg), and saponins (10 mg/kg) significantly reduced the early and late phases of the formalin nociceptive reaction compared to the negative control (Figure 5).

### 2.5. Molecular Docking of the Main Phenolic Compounds of C. europaea Polyphenolic Extract

CK1 and GSK3B are two proteins that play a crucial role in the activation of the Wnt/β-catenin signaling pathway and, therefore, the acceleration of wound healing. The CK1 family members share a common topology, consisting of an N-terminal regulatory domain, a central kinase domain, and a C-terminal tail. The kinase domain is responsible for the catalytic activity of CK1 and contains the binding site for ATP and the substrate. The N-terminal regulatory domain contains various phosphorylation sites, which can modulate the activity of CK1, and also serves as a binding site for regulatory proteins. The binding site of CK1 is located in the kinase domain and is composed of several conserved amino acid residues, including the ATP-binding site and the substrate-binding site. The ATP-binding site is composed of several key residues, such as the catalytic lysine, which is responsible for the phosphotransfer reaction, and the Asp-Phe-Gly (DFG) motif, which is involved in the regulation of the enzyme’s activity. The substrate-binding site is composed of several hydrophobic and basic residues that interact with the substrate [14].

The topology of GSK3B is an alpha/beta protein kinase fold, a common fold for many protein kinases, and it is composed of two lobes, the N-lobe and C-lobe. The N-lobe contains the binding site for ATP, and the C-lobe contains the binding site for the protein substrate. The two lobes are connected by a hinge region, which allows the two lobes to move relative to each other during the catalytic process.

The binding site of GSK3B is located within its kinase domain, and it specifically binds to the phosphorylated serine or threonine residues on its target proteins. The binding site is composed of a number of amino acid residues that form a pocket, which is responsible for recognizing and binding to the phosphate group on the target protein. Some compounds that have been reported to bind to the GSK3B binding site include certain small molecules and peptides, as well as larger protein complexes [15].

The phytocompounds identified in the *C. europaea* polyphenolic extract show a very important activity on these two proteins. Concerning the binding energy expressed by the glide score, luteolin 7-*O*-glucoside, isorhamnetin-3-*O*-rutinoside, amentoflavone, kaempferol-3-*O*-glucoside presented a very high binding energy with CK-1 of −5.949, −5.171, −4.679, −3.021 kcal/mol, respectively. Regarding the GSK-3β receptor, luteolin 7-*O*-glucoside, amentoflavone, luteolin, quercetin, and hesperetin showed the highest glide score energies of −7.696, −7.335, −7.258, −7.236, −6.838 kcal/mol, respectively.

Cyclooxygenase-2 (COX-2) is a membrane-bound enzyme that catalyzes the production of prostaglandins and thromboxanes from arachidonic acid. The enzyme is composed of two identical subunits, each of which contains a binding site for arachidonic acid and a catalytic site for the conversion of arachidonic acid to prostaglandins. The binding site for arachidonic acid is located within a hydrophobic pocket on the enzyme and is composed of amino acid residues from both the N- and C-terminal domains of the enzyme. The topology of COX-2 is such that the active site is located at the interface between the two subunits [16]. Moreover, in analgesic activity, trimethoxyflavone, luteolin, quercetin, and hesperetin exhibited the strongest binding energy in the active site of cyclooxygenase-2 with the energy of −8.572, −8.431, −7.749, and −7.657 Kcal/mol, respectively. By contrast, paracetamol and tramadol presented a glide energy score of −5.644 and −6.710 Kcal/mol (Table 3).

The tyrosine kinase plays a very important role in antileukemic activity. Tyrosine kinases are a family of enzymes that transfer a phosphate group from ATP to a tyrosine residue on a protein substrate. The kinase domain of these enzymes is composed of several structural regions, including an N-terminal regulatory domain, a central catalytic domain, and a C-terminal regulatory domain. The binding site for the protein substrate is located within the catalytic domain and is composed of several amino acid residues that form specific interactions with the substrate. These interactions include hydrogen bonds, hydrophobic interactions, and electrostatic interactions. The binding site is also highly specific and typically only recognizes one or a few specific substrate proteins [17]. The molecular docking of all the molecules identified in *C. europaea* in the active site of this protein showed a remarkable activity of hesperetin, quercetin, and luteolin with the energy of bindings of −9.187, −8.687, and −8.464 Kcal/mol, respectively.

Furthermore, the apoptotic effect of our plant was evaluated using the TRADD (TNF receptor-associated death domain protein). It is a signaling protein that is involved in the cellular response to TNF (tumor necrosis factor) and other cytokines. It is a cytosolic protein that contains a death domain, which is a protein–protein interaction motif that is important for mediating cell death signaling. The binding site of TRADD is located in the death domain, and it binds to the death domain of other proteins, such as the TNF receptor 1 (TNFR1) and Fas. This binding initiates a cascade of signaling events that ultimately leads to the activation of caspases, enzymes that are responsible for programmed cell death (apoptosis). The binding of TRADD to TNFR1 and Fas is mediated by the specific amino acid residues present in the death domain of these proteins. TRADD also contains a RING finger domain that is involved in ubiquitination, which is a process that targets proteins for degradation by the proteasome. The RING finger domain of TRADD can interact with other proteins that also contain RING finger domains, such as the TNF receptor-associated factor (TRAF) proteins, to mediate this process [18]. Isorhamnetin-3-*O*-rutinoside, luteolin, and quercetin showed an energy binding of −7.416, −6.885, and −6.312 Kcal/mol, respectively, against TRADD protein. Obatoclax (positive control) presented a glide energy score of −5.055 Kcal/mol (Table 3).

**Figure 6:**
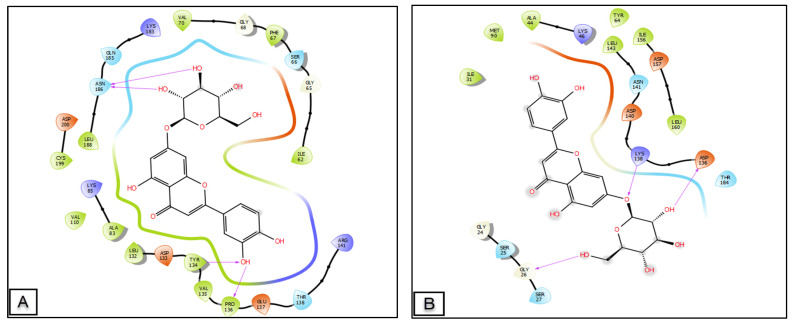

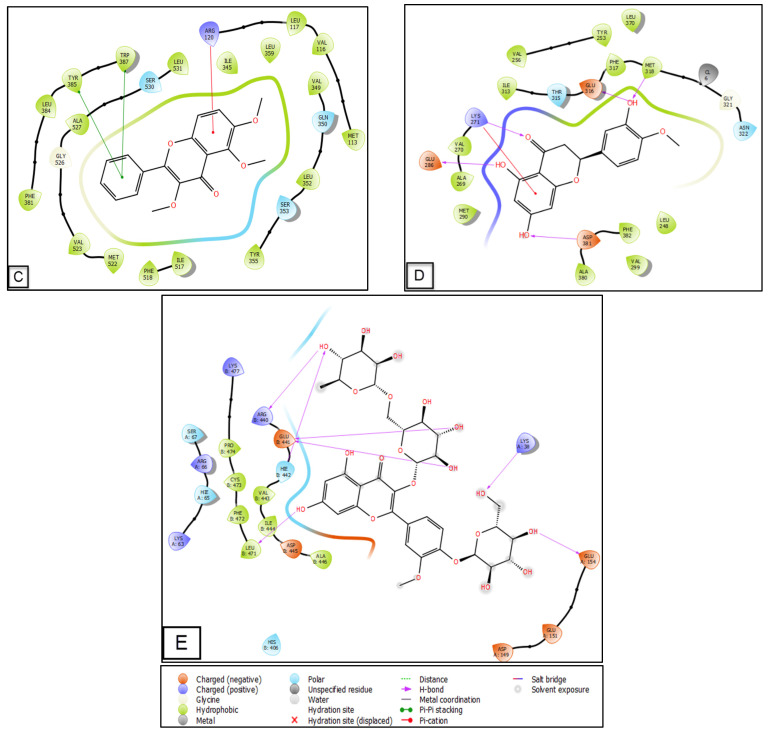
2D diagrams of candidate ligand interactions with the active sites. (**A**,**B**) luteolin 7-*O*-glucoside interactions with the active site of GSK3-β and CK-1, respectively. (**C**) Trimethoxyflavone interactions with the active site of cyclooxygenase-2. (**D**) Hesperetin interactions with the active site of tyrosine kinase. (**E**) isorhamnetin-3-*O*-rutinoside interactions with the active site of TRADD.

**Figure 7:**
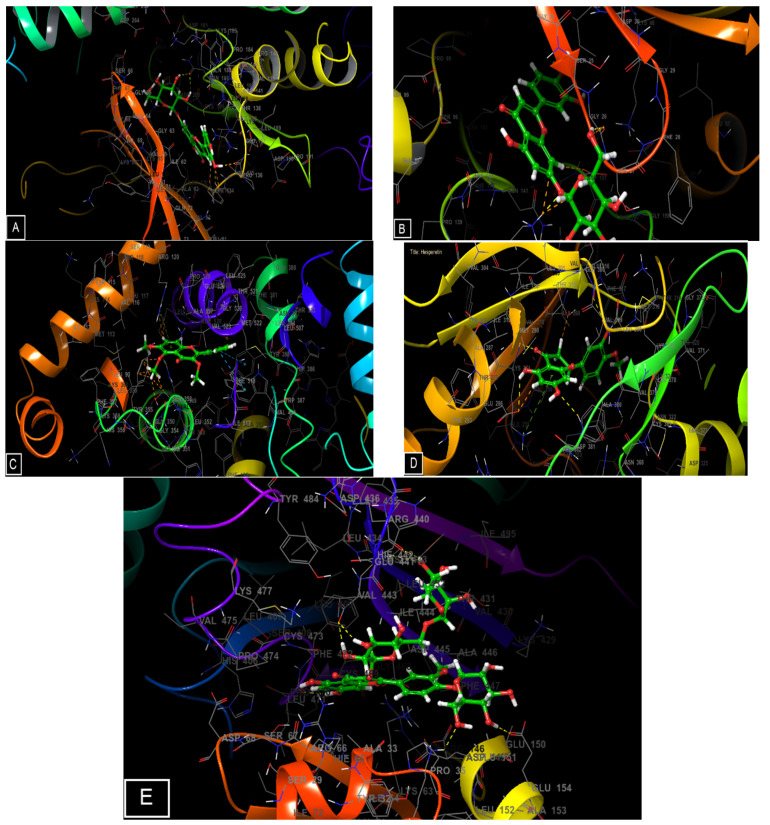
3D diagrams of candidate ligand interactions with the active sites. (**A**,**B**) luteolin 7-*O*-glucoside interactions with the active site of GSK3-β and CK-1, respectively. (**C**) Trimethoxyflavone interactions with the active site of cyclooxygenase-2. (**D**) Hesperetin interactions with the active site of Tyrosine kinase. (**E**) isorhamnetin-3-*O*-rutinoside interactions with the active site of TRADD.

Figure 6 and Figure 7 show the number and types of possible bonds between the ligands in the active sites. Luteolin 7-*O*-glucoside established four hydrogen bonds with residues ASN 186 (distance: 1.95 Å and 2.55 Å, angle: 150.1° and 144.4°), TYR 134 (distance: 1.84 Å, angle: 139.1°), and PRO 136 (distance: 1.78 Å, angle: 141.7°) in the GSK3-β receptor, and also three hydrogen bonds with residues LYS 138 (distance: 1.81 Å, angle: 177.3°), ASP 136 (distance: 1.73 Å, angle: 132.8°), and GLY 26 (distance: 1.76 Å, angle: 134.3°) in the CK-1 receptor. Trimethoxyflavone established one Pi-cation with residue ARG 120 and 2 Pi-Pi stacking with residues TYR 385 and TRP 387 in the active site of cyclooxygenase-2. In the active site of TRADD, isorhamnetin- 3-*O*-rutinoside established seven hydrogen bonds with residues of GLU 141 (distance: 2.11 Å and 1.75 Å, angle: 91.8° and 150.6°), ARG 440 (distance: 2.44 Å, angle: 116.6°), GLU 154 (distance: 1.61 Å, angle: 164.3°), LYS 38 (distance: 2.33 Å, angle: 125.9°), HIS 442 (distance: 2.29 Å, angle: 164.2°), and LEU 471 (distance: 1.75 Å, angle: 129.8°), while Obatoclax established a single hydrogen bond with residue GLN 143 (distance: 2.02 Å, angle: 154.4°) and a Pi-Pi stacking bond with residue PHE 410.

## 3. Discussion

*C. europaea* extracts have long been used in Moroccan traditional medicine. However, to date, there is no data reporting on the burn-healing, analgesic, or cytotoxic activities of *C. europaea* on human leukemia and hepatocellular carcinoma cell lines. Our work aims to draw attention to the application of *C. europaea* extracts as an alternative therapy for wound healing, pain, human leukemia, and hepatocellular carcinoma.

Therefore, we evaluated the effects of the *C. europaea* extracts on human leukemia and hepatocellular carcinoma cell lines, pain, burns, and the potential effect of the natural components identified in *C. europaea* polyphenols, to inhibit CK1 and GSK3B implicated in the Wnt/β-catenin signaling pathway, tyrosine kinase associated to leukemic cancer, and cox-2 implicated in the analgesic effect.

The phytochemical composition of plants can differ between species and origins. The technique, and the solvents used for extraction, can also have a significant impact on the chemical composition, and the biological activities of plants [19]. The UHPLC analysis of *C. europaea* polyphenolic extract revealed the presence of flavonoids (kaempferol, luteolin and kaempferol-3-*O*-hexose deoxyhexose) and phenolic acids (trans-ferulic acid, and syringic acid). These phytochemicals explain the healing, antinociceptive, and cytotoxic effects of the plant.

The wound healing process is divided into three main stages: (i) inflammation, (ii) proliferation, and (iii) remodeling. During the first phase, the Wnt/β-catenin signaling pathway begins and there is an influx of inflammatory cells. During the second phase, the wound is re-epithelialized due to the increase in the local Wnt response and the accumulation of β-catenin in the cytoplasm. An increase in β-catenin leads to gene transcription of matrix metalloproteinases, which promotes the growth of numerous cell types such as fibroblasts and keratinocytes and causes extracellular matrix deposition and angiogenesis. The third phase is characterized by significant remodeling of the extracellular matrix [20].

Several phenolic compounds such as kaempferol, luteolin, and ferulic acid are known to possess an important wound healing effect. Kaempferol is a flavanol with antioxidant and anti-inflammatory properties. Kaempferol exerted its healing activities by enhancing wound tensile strength, raising the amount of collagen and hydroxyproline in the wound, aiding wound closure, and accelerating re-epithelialization [21].

Luteolin is a phytochemical that modulates many inflammatory and antioxidant processes. It can suppress pro-inflammatory mediators such as IL-6, IL-8, TNF-α, and COX-2 and control a number of signaling pathways including NF-κB, and TLR [22]. Ferulic acid inhibited lipid peroxidation and elevated catalase, superoxide dismutase, reduced glutathione and nitric oxide (NO) levels, which reduce the wound areas. NO is known for its (i) stimulating effect on cell proliferation, (ii) angiogenesis, (iii) regeneration, (iv) increasing fibroblast proliferation and collagen production in wound healing [23].

The Wnt/β-catenin signaling pathway has been reported to play an important role in the repair of skin lesions. It is directly involved in restoring healing via the activation of dermal fibroblasts and keratinocytes proliferation [24]. The ineffective effect of the Wnt/β-catenin signaling pathway leads to poor wound healing [25].

The Wnt/β-catenin signaling pathway involves three key phases: (i) transduction of Wnt signaling in the membrane, (ii) regulation of β-catenin stabilization in the cytoplasm and (iii) stimulation of gene transcription, cell division, and cell proliferation caused by an accumulation of β-catenin in the nucleus.

When Wnt ligands are absent, a proteasome complex of CK1, GSK-3, axin, and APC breaks down β-catenin. However, in the presence of Wnt ligands, the interaction of Wnt and receptors dissociates the proteasome, which causes the accumulation of β-catenin in the nucleus and promotes the expression of target genes [26].

In the Wnt/β-catenin signaling pathway, GSK3-β and CK-1 phosphorylate β-catenin have two roles, by acting as activators and inhibitors of Wnt [27]. The binding of ligands to targets could enhance their activity, which may increase the wound healing process, while phenolic molecules identified in *C. europaea* polyphenols were docked against GSK3-β and CK-1 of the Wnt/β-catenin pathway (Figure 6).

Luteolin 7-*O*-glucoside, isorhamnetin-3-*O*-rutinoside, amentoflavone, and kaempferol-3-*O*-glucoside presented a very high binding energy with CK1. Regarding the GSK3-β receptor, luteolin 7-*O*-glucoside, amentoflavone, luteolin, quercetin, and hesperetin showed the highest glide score energies (Table 3).

Previous works demonstrated that quercetin accelerated the healing of cutaneous wounds by promoting fibroblast migration and proliferation, decreasing inflammation, and upregulating the production of growth factors via the Wnt/β-catenin signaling pathway [28].

The molecular docking of *Lavandula officinalis* phytocompounds showed a glide score energy between −4.596 and −5.34 kcal/mol in the active site of casein kinase-1, and a glide score energy between −6.298 and −7.652 kcal/mol in the active site of glycogen synthase kinase-3β [29]. Another study targeted CK1 in the same active site, hence the importance of this active site to target this protein [30].

The antinociceptive activity of the *C. europaea* extracts was evaluated using two methods: the writhing test conducted to examine the peripheral antinociceptive effect, and the formalin test used to explore peripheral and central mechanisms [31].

Peripheral antinociceptive activity may be mediated through the inhibition of lipoxygenases and/or cyclooxygenases and other inflammatory mediators [32]. Compared to the positive control, all extracts of *C. europaea* showed significant suppression of writhes in animals (Figure 4).

The formalin test was used to confirm the antinociceptive effect of the *C. europaea* extracts. Injection of formalin induces a biphasic nociceptive reaction manifested by biting and licking the injected paw. The early phase is caused by the direct action of formalin on nociceptors, which lasts for 0–5 min, and the late phase lasts from 15 to 30 min after formalin injection, mediated by a combination of spinal cord sensitization and peripheral input [33,34].

Oral administration of hydroethanolic, polyphenols, flavonoids, and saponins extracts of *C. europaea* at the doses of 100, 50, 15, and 10 mg/kg, respectively, significantly reduced the amount of time spent licking the injected paw in both phases (Figure 5). These results are in accordance with a previous study reporting that pretreatment with ethanolic extract of *C. europaea* at the doses of 100 mg/kg and 200 mg/kg significantly reduced both phases of formalin nociceptive response [31].

These findings demonstrated that the *C. europaea* extracts act both peripherally and centrally. The antinociceptive effect of *C. europaea* may be related to its phenolic compounds such as vanillic acid, ferulic acid, myricetin, quercetin, and naringenin [35].

The active site of cyclooxygenase-2 (COX-2) is the region within the protein where substrate molecules bind and undergo chemical reactions. This site is crucial for the catalytic activity of COX-2. A previous study targeted the same active site of cyclooxygenase-2 studied in our research; in this active site, *Lavandula officinalis* molecules showed a glide score energy of −6.156 and −7.526 kcal/mol [29]. Other studies used the same residue-limited active site to evaluate phytochemicals derived from *Holigarna caustica* (Dennst.) and *Sterculia foetida* for pain management [36].

Analgesic drugs have been divided into three groups: non-opioid analgesics, weak opioids, and strong opioids. Non-opioid analgesics are essentially represented by non-steroidal anti-inflammatory (NSAI) drugs [37]. NSAI drugs act by inhibiting the cyclo-oxygenase-2 (COX-2), and therefore the central inhibition of prostaglandin synthesis responsible for pain [38]. Previous studies demonstrated that amentoflavone and luteolin suppresses COX-2 expression [39].

Abnormal tyrosine kinases activity is involved in various types of cancer and has inspired scientific researchers to develop tyrosine kinase inhibitor drugs as a form of cancer treatment. For most chronic myeloid leukemia patients, tyrosine kinase inhibitors have transformed a fatal disease into a manageable chronic illness [40]. Imatinib is a tyrosine kinase inhibitor used to treat chronic myeloid leukemia. Imatinib treatment of patients with early-stage chronic myeloid leukemia increased five-year survival rates from 40% to 50% to 90% [40].

Tyrosine kinase inhibitors play an important role in the treatment of chronic myeloid leukemia. However, several challenges limit the therapeutic value of these drugs such as intolerance, resistance to these inhibitors, and other negative side effects. Therefore, the discovery of natural antileukemia treatments with fewer side effects is a promising avenue for the prevention and treatment of leukemia. Phenolic compounds are among these natural treatments that have an interesting anti-cancer potential [41].

Flavonoids have been studied for their antitumoral effect against several cancer cell lines and cancers [42]. They have shown numerous biological characteristics that are associated with chemopreventive effects and lower leukemia cancer rates [43]. Quercetin, luteolin, and hesperetin, naturally occurring flavonoids, have been reported as powerful antioxidants with chemotherapeutic effects against leukemic cancer.

Quercetin induced apoptosis of K 562 cells, inhibited K 562 cells proliferation, and improved Bax, caspase-3, and caspase-8 expression in the K-562 cell lines [44]. Luteolin has the potential to be a dietary anticarcinogenic drug by inhibiting tyrosine kinase effects, an enzyme involved in the proliferation of tumor cells. It has been proposed that the mitochondrial pathway plays a significant role in the ability of luteolin to cause apoptosis in HL-60 cells [45]. In chronic myeloid leukemia cells, hesperetin reduced cell growth and induced apoptosis of K562 [41].

The active site of tyrosine kinase is involved in antileukemic activity through its role in regulating cell survival. By targeting the active site of tyrosine kinase, antileukemic drugs can help to control the progression of leukemia [46]. A molecular docking study evaluating the effect of benzyl 3-hydroxy-23-oxoolean-12-en-28-oate against tyrosine kinase was evaluated in the same active site used in our study and showed a bond formation with the same residues found in our study. These results show the importance of using this active site as a drug target [47].

The extrinsic apoptotic pathway involves membrane death receptors. Many death receptors have been identified, including TNF-R1 (Tumor Necrosis Factor Receptor 1) and Fas. In the case of the TNF receptor, ligand activation causes the recruitment of an intracellular adaptor molecule called TRADD at the receptor site. TRADD is an adaptor protein that interacts with another death domain-containing molecule FADD (Fas-associated death domain protein). Effector caspases such as caspase 3 are activated as a result of interactions between pro-caspases and FADD, and then the apoptotic signal is sent to the intracellular death substrates [48]. TNF-α binding to TNFR1 can mediate cell death through the binding of TRADD. A high concentration of TRADD increased the level of pro-apoptotic molecules [49].

TRADD (TNF receptor-associated death domain protein) acts as a scaffold protein that recruits other proteins to the death-inducing signaling complex (DISC), which is formed upon activation of the TNF receptor. The death domain (DD) of TRADD is the active site responsible for the recruitment of other proteins to the DISC and for the initiation of apoptosis [50]. A study presented the same active site used in our study with the same residues as the active site to target this protein [51].

## 4. Materials and Methods

### 4.1. Plant Material

**Figure 8:**
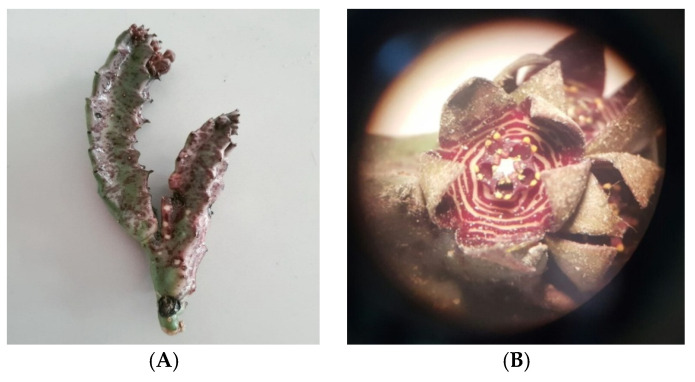
*C. europaea* aerial parts: (**A**) Stem; (**B**) Flower.

The aerial part of *C. europaea* was harvested in the spring of 2021 in the Imouzzer mountains of the Moroccan Middle Atlas (30°40′48″ N 9°28′58″ W). Botanist Amina Bari authenticated the plant under the voucher specimen “18I4C001”. The plant name was verified using the online database “The Plant list” (Figure 8). *C. europaea* was chosen based on our previous ethnopharmacological study in Fez-Meknes region [52].

### 4.2. Animal Material

Adult female Swiss albino mice and male Wistar rats aged 2 months were provided from the animal house at the University Sidi Mohamed Ben Abdellah in Fez. The animals were housed in a controlled environment, with light and dark cycles of 12 h, and a temperature of 23 ± 2 °C. They had free access to food and water as well. The ethical guidelines for the handling and use of laboratory animals were followed during every animal experiment [53].

### 4.3. Preparation of C. europaea Extracts

#### 4.3.1. Hydroethanolic Extract

*C. europaea* powder (10 g) was extracted with 100 mL of ethanol (70%) using the ultrasonic device (SB100DT) for 45 min at 25 °C. The obtained mixture was filtered through Wathman paper, then condensed using a rotary evaporator (BUCHI:461) at 40 °C [9]. The yield of hydroethanolic extract was 13.25%.

#### 4.3.2. Polyphenolic Extract

*C. europaea* powder (10 g) was macerated with methanol (3 × 30 mL). The mixture was filtered and concentrated, and then dissolved in 50 mL of distilled water. The obtained solution was extracted three times with 20 mL of hexane, chloroform, and ethyl acetate solvents; the ethyl acetate phase was concentrated using a rotary evaporator to obtain polyphenolic fraction [10]. The yield of polyphenolic extract was 7%.

#### 4.3.3. Flavonoids Extract

Thirty grams of the *C. europaea* powder was macerated with 100 mL of methanol for 72 h at room temperature. The mixture was filtered and evaporated at 40 °C. The obtained residue was dissolved in distilled water (50 mL), and re-extracted with 3 × 30 mL of chloroform, diethyl ether, *n*-hexane, ethyl acetate, and *n*-butanol successively. The alcoholic phase containing the flavonoids was recorded, and then evaporated under a vaccum using a rotary evaporator at 40 °C [9]. The yield of flavonoids extract was 1.97%.

#### 4.3.4. Saponins Extract

Twenty grams of *C. europaea* powder was defatted with 200 mL of hexane for 2 h. After removal of organic phase and evaporation of the solvent, the obtained residue was macerated with 600 mL of ethanol for 24 h. The alcoholic phase was evaporated at 40 °C, and then extracted three times with 200 mL of distilled water/hexane (*v*/*v*). The aqueous phases were mixed and then taken up in 300 mL of n-butanol for 30 mn. The organic phase containing the saponins was evaporated at 40 °C [9]. The yield of saponins extract was 2.44%.

### 4.4. Phytochemical Composition of C. europaea Polyphenolic Extract

#### 4.4.1. Solvents and Reagents

Quercetin-3-*O*-glucuronic acid, kaempferol-3-*O*-glucuronic acid, ferulic acid, syringic acid SIM, quercetin SIM, isorhamnetin-7-*O*-pentose, quercetin-3-*O*-glucoside, trans-cinnamic acid, gallic acid SIM, trans-ferulic acid SIM, kaempferol-3-*O*-pentose, hesperetin, trimethoxyflavone, luteolin, kaempeferol, syringic acid, tyrosol, kaempferol-3-*O*-hexose deoxyhexose, luteolin-7-*O*-glucoside, isorhamnetin-3-*O*-rutinoside, Quercetin-3-*O*-hexose deoxyhexose, kaempferol-3-*O*-glucose, as well as amentoflavone were purchased from Sigma Aldrich (Hamburg, Germany).

#### 4.4.2. Analytical Equipment

The chemical analysis of *C. europaea* polyphenols was evaluated using Ultra High Performance Liquid Chromatography (UHPLC, Nexera, XR LC 40), coupled to a MS/MS detector (LC-MS 8060, Shimadzu). The flow of nebulizer gas was 2.9 L/min, the flow of dryer gas was 10 L/min, and the flow of heater gas was 10 L/min. The interface temperature was 300 °C, the DL temperature was 250 °C, and the heater block temperature was 400 °C. The MS/MS was operated with electrospray ionization (ESI+, and ESI-). The chromatographic separation was performed on a C18 Accucore Polar Premium column (2.6 µm; 46 × 150 mm), the mobile phase was a mixture of acetonitrile and water (5:95/*v*:*v*), in addition to formic acid (0.01%), the total run time was 5 min, and the flow rate was 0.7 mL/min in isocratic conditions. The database of phenolic compounds was used for the analysis of the obtained results based on the detection of the precursor ion (*m*/*z*) [54]. The polyphenols extract was dissolved in 1 mL of saline phosphate buffered acetone solution. After centrifugation, the supernatant was used for UHPLC analysis.

### 4.5. Cytotoxicity Assay of C. europaea Extracts

The cytotoxicity of the *C. europaea* extracts was evaluated using the MTT test (National Institute of Amazonian Research, Brazil). Human acute promyelocytic leukemia (HL60: ATCC^®^ CCL-240^TM^), human chronic myelogenous (K562; ATCC^®^ CCL-243^TM^), and human hepatocarcinoma (Huh-7) cell lines were cultured into a 96-well plate (2 × 10^4^ per well), containing 0.2 mL of RPMI medium per well, at 37 °C and 5% of CO_2_, for 24 h. After the formation of sub-confluent monolayer, the cell lines were exposed to various concentrations of the *C. europaea* extracts before being incubated for 24 h, 48 h and 72 h. Negative and positive controls were sterile PBS and DMSO 100%, respectively. The medium was then removed from each well, and 10 µL of diluted MTT in DMEM medium was added into the wells and incubated for 4 h under the same conditions as before. Thereafter, the MTT was removed and 50 µL of MTT lysis buffer was added to all wells, and the mixture was then incubated at 37 °C for 10 min. The sample’s absorbance was evaluated at 570 nm. The cell survival was determined as follows: A 570 nm of treated sample/A 570 nm of untreated sample × 100.

### 4.6. Wound Healing Activity of C. europaea Extracts

#### 4.6.1. Ointment Preparation

Ointments of the *C. europaea* extracts (10%) were prepared by melting 1 g of the hydro-ethanolic extract, polyphenols, flavonoids, and saponins in 9 g of Vaseline^®^ (*w*/*w*). Extracts were added to Vaseline^®^ in a beaker over a water bath at 50 °C, with continuous stirring until homogenous. Ointments were kept at 4 °C in airtight containers. The positive and negative controls were Madecassol^®^ 1% and Vaseline^®^ [55]. Madecassol^®^ 1% is a hydrocotyl ointment (1%), derived from the dry extract of *Centella asiatica*, used in the local treatment of skin wounds [56].

#### 4.6.2. Burn Wound Induction

Male Wistar rats weighing between 250, and 300 g were divided into six groups (*n* = 5). After anesthesia of test animals intraperitoneally with sodium pentobarbital at a dose of 50 mg/kg, the animals’ dorsal regions were shaved, and then burn wounds were made using a burn set with an aluminum rod (1.5 cm) that had been heated to 100 °C. Treatment started 24 h following burn wound induction. Ointments were applied daily for 14 days to the entire area of the wound. The burned areas of all animals groups were photographed using a ruler as a scale. At the end of the study, images of each day were evaluated using ImageJ software to determine the wound closure percentage [55].

The following formula was used to analyze photographs of the burned area and determine the rate at which wounds were contracting:

$$WC\;\left(\%\right)=\frac{\left(W_{S0}-WS_{SD}\right)}{W_{S0}}\;\times \;100$$

where WC (%): Wound contraction (%); W_S0_: Wound size on induction day; WS_SD_: Wound size in a specific day.

### 4.7. Antinociceptive Tests of C. europaea Extracts

#### 4.7.1. Acetic Acid Test

The antinociceptive activity of the *C. europaea* extracts was performed by calculating the number of abdominal contortions induced in mice by intraperitoneal injection of an aqueous solution of acetic acid (0.7%) at the dose of 10 mL/kg of the body weight. Oral treatment of mice by the tested extracts was carried out one hour before the injection of acetic acid. The writhes number of each mouse was counted for 30 min after intraperitoneal injection of acetic acid [57].

Thirty mice were divided into six groups (*n* = 5) and treated as follows:

- Group 1: Negative control (0.9% NaCl);
- Group 2: Paracetamol (100 mg/kg);
- Group 3: Hydroethanolic fraction (100 mg/kg);
- Group 4: Polyphenols fraction (50 mg/kg);
- Group 5: Flavonoids fraction (15 mg/kg);
- Group 6: Saponins fraction (10 mg/kg).

The inhibition percentage (IP) of the nociceptive response was calculated as follows [31]:

$$IP=\frac{\left(NC-NT\right)}{NC}\times 100$$

where NC: Number of contortions of the control group; NT: Number of contortions of treated groups.

#### 4.7.2. Formalin Test

The antinociceptive effect of the *C. europaea* extracts was evaluated by injecting 20 µL of formalin (2%) subcutaneously into the right posterior paw of the mice. The time spent licking the injected paw was considered as indicative of nociceptive behavior. The quantification of nociceptive behavior consists of two successive phases: the early phase response peaked 5 min after formalin injection and the late response peaked 15–30 min after formalin injection [31]. The animals were pre-treated orally one hour beforehand as follows:

- Group 1: Negative control (0.9% NaCl);
- Group 2: Positive control (Tramadol);
- Group 3: Hydroethanolic extract (100 mg/kg);
- Group 4: Polyphenols extract (50 mg/kg);
- Group 5: Flavonoids extract (15 mg/kg);
- Group 6: Saponins extract (10 mg/kg)

### 4.8. Molecular Docking

We have studied in this molecular docking investigation the different activities of all phytocompounds revealed in *C. europaea* polyphenolic extract, including healing activity (casein kinase-1 and glycogen synthase kinase-3β), analgesic activity (cyclooxygenase-2), antileukemic activity (Tyrosine kinase), and apoptotic effect (TRADD).

The SDF format of phenolic compounds identified in the polyphenolic extract of *C. europaea* and molecules used as a positive control was retrieved from the PubChem database. Then, they were prepared for molecular docking using the OPLS3 force field and the LigPrep tool in the Maestro 11.5 release of the Schrödinger Software. In all, 32 stereoisomers were generated for each ligand, taking into account ionization states at pH 7.0 and 2.0.

The crystal structures of CK1, GSK3, cyclooxygenase-2, tyrosine kinase, and TRADD were obtained from the Protein Data Bank in PDB format using the designated PDB IDs: 6GZD, 1Q5K, 6COX, 1IEP, and 1F3V, respectively. The Schrödinger-Maestro v11.5 software was used to prepare and construct the structures. Selenomethionines were converted into methionines, heavy atoms were given hydrogens, and all waters were removed. Charges and bond ordering were also allocated. The maximum heavy atom RMSD (root-mean-square-deviation) was set to 0.30 during minimization using the force field OPLS3.

The process of generating the receptor grid starts by selecting any atom of the ligand, and the grid is spaced at 20 × 20 × 20 intervals. The non-cis/trans-amide bond was penalized during SP flexible ligand docking in Glide of Schrödinger (Maestro v 11.5). The partial charge cutoff and van der Waals scaling factor were set at 0.15 and 0.80, respectively. The final score, known as the glide score, was determined based on the most energy-efficient positions. The ligand’s best-docked position with the lowest glide score value was recorded for each ligand [54].

### 4.9. Statistical Analysis

Statistical analysis was performed using One-way ANOVA, analysis of variance with GraphPad Prism (GraphPad software, La Jolla, CA, USA). The results were expressed as means ± SEM from at least three independent experiments. * *p* value < 0.05 was considered as statistically significant.

## 5. Conclusions

The anticancer activity of *C. europaea* extracts shows the possibility of using *C. europaea* clinically as a potent antileukemic agent and inhibitor of hepatocellular carcinoma. All *C. europaea* extracts have significant analgesic activity. The topical application of all *C. europaea* formulations had important healing effects on the wounds in rats. The phenolic compounds such as luteolin 7-*O*-glucoside, isorhamnetin-3-*O*-rutinoside, and kaempferol-3-*O*-glucoside appear to be promising agents for the treatment of wounds by inhibiting the Wnt/β-catenin signaling pathway. Further clinical trials are required for optimization/validation of the extract’s use for therapeutic treatments.

## Figures and Tables

**Table 1:** Phenolic compounds identified in the *C. europaea* polyphenolic extract.

Polyphenolic Extract		
Molecule	AUC	*m*/*z*
Gallic acid	83,057	168.9
Quercetin	4,260,116	301
Trans-ferulic acid	478,543	193
Hesperetin	6,639,841	301.3
Amentoflavone	325,795	537.1
Luteolin	69,281,031	284.9
Kaempferol-3-*O*-glucose	1,458,547	609.1
Quercetin-3-*O*-hexose deoxyhexose	1,673,879	609.1
Isorhamnetin-3-*O*-rutinoside	1,432,451	623.1
Isorhamnetin-7-*O*-pentose	8,344,809	447.1
Luteolin 7-*O*-glucoside	8,266,394	447.1
Kaempferol-3-*O*-glucuronic acid	1,007,610	461.1
Kaempferol-3-*O*-pentose	1,665,472	417.1
Kaempferol-3-*O*-hexose deoxyhexose	19,807,364	593.1
Tyrosol	491,809	153.4
Syringic acid	365,324	198.9
Ferulic acid	214,942	193
Kaempferol	102,866,891	285

**Table 2:** Anticancer activity of the *C. europaea* extracts.

*C. europaea* Extracts	IC_50_ (µM)		
	Human Chronic Myelogenous Leukemia (K562 Cell Line)	Human Acute Promyelocytic Leukemia (HL60 Cell Line)	Human Hepatocellular Carcinoma (Huh-7 Cell Line)
Hydroethanolic	>100	>100	>100
Polyphenols	36.47 ***	53.77 ***	24.07 ***
Flavonoids	>100	>100	>100
Saponins	>100	>100	50.14 ***

*** Activity observed only during the 72 h of treatment.

**Table 3:** Docking results with ligands in Casein kinase-1, Glycogen synthase kinase-3β, cyclooxygenase-2, Tyrosine kinase, and TRADD receptors.

	Glide G-Score (Kcal/mol)				
	Wound Healing Effect		Analgesic Activity (Cyclooxygenase-2)	Antileukemic Activity (Tyrosine Kinase)	Anti-Hepatocellular Carcinoma (TRADD)
	Casein Kinase-1	Glycogen Synthase Kinase-3β			
Luteolin 7-*O*-glucoside	−5.949	−7.696	-	-	−5.977
Amentoflavone	−4.679	−7.335	-	-	-
Trimethoxyflavone	-	-	−8.572	−8.304	−3.975
Luteolin	-	−7.258	−8.431	−8.464	−6.885
Quercetin	-	−7.236	−7.749	−8.687	−6.312
Hesperetin	-	−6.838	−7.657	−9.187	−5.03
Kaempferol	-	−6.716	−7	−7.964	−6.569
Ferulic Acid	-	−6.669	−6.156	−6.692	-
Kaempferol-3-*O*-glucoside	−3.345	−6.449	-	-	-
Isorhamnetin-3-*O*-rutinoside	−5.171	−6.196	-	−7.47	−7.416
Tyrosol	-	−5.174	−6.141	−6.925	−4.149
Syringic acid	-	−5.047	−6.404	−6.075	−4.794
Gallic acid	-	−4.997	−5.894	−6.751	−4.542
Paracetamol	NA	NA	−5.644	NA	NA
Tramadol	NA	NA	−6.710	NA	NA
Obatoclax	NA	NA	NA	NA	−5.055

NA: Not Applicable.

## Data Availability

Not applicable.
